# Perceived role of the veterinarian in promoting dairy cattle welfare

**DOI:** 10.3389/fvets.2023.1325087

**Published:** 2023-12-18

**Authors:** Michael W. Brunt, Derek B. Haley, Stephen J. LeBlanc, David F. Kelton

**Affiliations:** ^1^Department of Population Medicine, Ontario Veterinary College, University of Guelph, Guelph, ON, Canada; ^2^Campbell Centre for the Study of Animal Welfare, University of Guelph, Guelph, ON, Canada; ^3^Dairy at Guelph, University of Guelph, Guelph, ON, Canada

**Keywords:** mixed-methods, cross-sectional, valanced experiences, bovine, veterinary student, prioritize

## Abstract

Improving the lives of animals in agricultural systems has primarily focused on minimizing negative experiences. Research is needed on the promotion of positive experiences (pleasure, enjoyment, play, choice, happiness) for animals and the role of veterinarians in their promotion. Our aims were to describe how Canadian bovine veterinarians and veterinary students perceive the role of a veterinarian in positive vs. negative experiences for dairy cows and to analyze the rationale provided to explain their answers. Canadian veterinary practitioners (*n* = 78) and veterinary students (*n* = 148) responded to an online cross-sectional survey and were asked, on a 7-point scale, how important the role of a veterinarian is to promote practices that influence the experience of dairy cows. We used qualitative description to analyse participants’ open-ended text responses. Practices to minimize negative experiences were most important (mean ± SE; 6.8 ± 0.03), a balance of positive and negative experiences was less important (6.4 ± 0.05), and encouragement of positive experiences scored lowest (6.0 ± 0.06), although all scored highly. Four themes were identified to explain participants’ reasoning regarding their perceived role of a veterinarian in the promotion of dairy cattle welfare, centered on: the animal, the producer, the veterinarian, and society. Participants indicated that promoting positive experiences was less important than decreasing negative experiences (5.9 ± 0.09). There were four themes identified to explain participants’ reasoning regarding the relative importance of promotion of positive experiences versus decreasing negative experiences which centered on: frameworks to compare positive and negative experiences, impacts on the animal, the participant’s view of their role, and the practicality of implementation. These results indicate modest differences in valuing avoidance of negative vs. promotion of positive welfare. There were no differences in the quantitative analyses between veterinarians and veterinary students. We conclude that veterinarians are favorably disposed to positive aspects of welfare for dairy cows but may be more focussed on avoidance of negative aspects of welfare.

## Introduction

The Canadian dairy industry comprises nearly 1.4 million cows and heifers cared for by farmers on over 9,700 farms (1). Improving the lives of animals in agricultural systems has primarily focused on minimizing negative experiences (2). Historically, dairy veterinarians have aimed to reduce pain through prompt diagnosis and treatment of diseases such as lameness or mastitis and more recently to minimize pain during routine procedures like dehorning (3, 4). The last 15 years has seen advancements in affective neuroscience resulting in enhanced understanding of animal experiences and has created opportunities for positive engagement with animals (5). Better understanding of the capacity for some animals to experience valanced (positive or negative) events moved ethical discussions beyond pain and distress (6, 7). Positive welfare is often described through two different views: “hedonic positive welfare” (an animal’s welfare improves because it gets what it likes and wants) and “positive welfare balance” (positive experiences outweigh negative experiences) (8). A critical review linked positive welfare to existing aspects of animal welfare research and emphasized new opportunities for collaboration (9). While global animal welfare is an ethically complex, multi-stakeholder topic, positive welfare can contribute to a more balanced approach in this area (10).

Research into the promotion of positive experiences (pleasure, enjoyment, play, choice, happiness) for animals is expanding (9). Cattle are highly motivated to access mechanical brushes (11) and readily utilize stationary brushes (12). They are also highly motivated to access pasture (13) and reunite with their calf (14). While calves are similarly highly motivated to access outdoor space (15) there is increased cognitive performance (16), play behavior (17), and decreased avoidance of new foods (18) when housed in social groups. Encouragement of producers by veterinarians to consider cattle brushes, calm animal handling, and additional autonomy (e.g., voluntary milking; feed and pasture access) could advance positive opportunities for dairy cattle.

Veterinarians are seen by dairy producers as persuasive stakeholders with the ability to influence animal welfare on dairy farms (19) and are key advisors in ensuring animal health (20, 21). While the diagnosis of illness is important in establishing the authority of veterinary professionals, they must also balance complex ethical responsibilities between their profession, the owner, and the animal patient (22). Producers’ decision-making on dairy farms is influenced by their veterinarians (23, 24). Farmers described the authority of veterinarians to extend beyond disease diagnosis and treatment and to include animal welfare (25). Given the influence of veterinarians on farm practices, it is important to understand what aspects of welfare these professionals perceive to fall within the scope of bovine veterinary medicine.

Veterinarians are often concerned with production disease diagnosis (26), disease treatment (27), and biosecurity on farms (28). One study found that Canadian dairy veterinarians prioritized the physical health of calves over other issues (e.g., social needs) (29). Research with veterinary students found they inconsistently applied welfare principles between production and companion animal species in the United States (30), China (31), Croatia (32), Australia and New Zealand (33). However, there has been little exploration of how veterinarians and veterinary students in Canada perceive their role in the promotion of positive welfare states.

We developed our approach in this study using the framework proposed by Rault et al. (8). Our objectives were to describe how Canadian bovine veterinarians and veterinary students perceive the role of a veterinarian in the promotion of dairy cattle welfare and to analyze the rationale provided to explain their answers.

## Materials and methods

### Participant recruitment and survey design

The study was approved by the University of Guelph Research Ethics Board (22-09-017) and is reported in accordance with the STROBE guidelines (34). Purposive theory-based sampling guided recruitment strategies during survey development (35). All veterinarians who were members of the Canadian Association of Bovine Veterinarians and veterinary students studying at colleges in Canada were invited twice to participate in the survey. Veterinarians were invited via emails sent by the Canadian Association of Bovine Veterinarians. All veterinary students at all veterinary colleges in Canada received the survey. Veterinary students at four colleges were invited to participate via email from their student government and students at the other college were emailed by the college administration. The survey opened on November 10, 2022, closed February 1, 2023, and took approximately 10 min to complete. The exploratory nature of this study did not necessitate the calculation of a target sample size. However, based on previous research (36, 37), a response rate of 3 to 5 % (*n* = 86 to 143) was expected.

The survey was available in French and English^1^. It was written in English and translated into French by a native French speaker and validated by a bilingual coauthor (SL). Participants accessed the survey through the cloud-based survey platform Qualtrics (version November 2022, Qualtrics). The survey was pre-tested for clarity by nine participants from the University of Guelph, but these were not included in the analysis. Participants were asked: “An important role of a veterinarian is,” followed by three sets of (total of nine) similarly worded statements, using a 7-point Likert scale, and to describe the reason for their response in a text box. The three sets of three statements described: (1) minimizing negative experiences, (2) encouraging positive experiences, or (3) encouraging a balance of positive and negative experiences.

The participants were then asked their level of agreement with the statement: “Overall, promoting positive experiences (e.g., natural behaviors, positive emotions, a good life) is as important as minimizing negative experiences (e.g., disease, pain, distress) for dairy cows.” Each of the seven Likert response options was labeled from strongly disagree to strongly agree. Participant attention checks were employed to account for random (first attention check) and non-random (second attention check) inattentive responses (38). The first was by asking the participants to answer “Disagree” to a specific question. The second reversed the Likert order of one question. Participants were excluded if they entered the same extreme value for all questions in this section (e.g., 2, 2, 2…) but the same intermediate values (i.e., 3, 4, 5) were considered realistic and included in the analysis. We also asked a series of demographic questions on variables (see below) associated with attitudes toward animals (39).

### Analysis

#### Quantitative data

Quantitative data were analyzed in SAS (version 9.04, SAS Institute Inc.). There was good internal consistency across the three statements about minimizing negative welfare (Cronbach alpha = 0.84), the three statements to promote hedonistic experiences (Cronbach alpha = 0.82), and the three statements about promoting a positive welfare balance (Cronbach alpha = 0.83). Factor analysis (FACTOR procedure in SAS) was used to assess the unidimentionality of these three constructs. Each construct only had one retained factor with eigenvalues >1 and in combination with visual inspections of Scree tests, the unidimensionality was confirmed (40, 41). Therefore, using the Likert responses, the mean of each statement grouping provided a minimizing negative experiences score, promoting positive experiences score, and a balanced positive vs. negative experience score (42). A linear regression model was used (MIXED procedures in SAS) to assess associations of participant demographic factors for the statement groupings. A linear regression model was used (GLM procedures in SAS) to assess associations of participant demographic factors for the relative importance of promoting positive experiences versus decreasing negative experiences. Demographic factors included gender (woman v. man plus gender not listed and prefer not to disclose), graduate training (yes v. no), years of veterinary practice experience (continuous), region (Atlantic v. Quebec v. Ontario v. Western), role (veterinarian dairy cows >50% of practice v. veterinarian dairy cows <50% of practice v. veterinary student), and the interaction between each significant demographic factor and the score for each outcome. To improve representativeness, least squares means were weighted based on the distribution of the categorical variables in the data. Demographic factors and interaction terms were removed from the final models if their *p*-value was >0.05, and we assessed the normality of model residuals.

#### Qualitative data

Qualitative description was used to analyze qualitative data (43). Participants could respond in French or English. The native language of the lead author was English and responses in French were translated with online translation software (Google Translate, Google LLC). The lead author coded a sample of 100 participant responses from each of the two open-ended questions (NVivo, version 12.7.0, QSR International Pty Ltd.). A codebook was developed for each of the two questions. Codes were identified through open coding, constant comparison, and axial coding before being amalgamated into themes (44). Inter-coder reliability and validity of both codebooks were established by the lead author and another researcher who independently coded a subset of 100 responses per codebook (45). There was substantial code agreement between the two researchers for the role of a veterinarian (Kappa = 0.77) and the relative importance of positive and negative experiences (Kappa = 0.76). Differences with coded responses were discussed until consensus was reached. All remaining participant responses were coded by the lead author with the final codebooks. The selection of quotations was based on how effectively they demonstrated the theme. Anonymous numbers were assigned to participants upon entry into the survey, preceded by the demographic identifiers of woman (W) or other (O) and veterinary student (S) or practitioner (V) and are associated with the quotes in the text. Any editing required for clarity is indicated using square brackets around inserted words.

## Result

The survey was started by 376 participants, representing a 13.1% response rate, with 254 completed surveys. The first and second attention checks were failed by 27 and 1 participants, respectively, resulting in 226 surveys included in the final analyses. Geographical breakdown of participant demographic characteristics is provided (Table 1). Veterinary practitioners had a mean age (years ± SD) of 45.0 ± 13.2, while veterinary students were 24.7 ± 3.3. Participants identifying as women made up nearly half of veterinarians (*n* = 38, 49%) and most of the veterinary students (*n* = 130, 88%). Graduate training (e.g., MSc, PhD, DVSc) was reported by 33% (*n* = 26) of veterinarians and 17% (*n* = 25) of veterinary students.

**Table 1:** Description of respondents to an online survey of veterinarians and veterinary students on the perceived role of a veterinarian in the promotion of positive welfare of dairy cows.

	Canada (total)		Atlantic provinces^1^		Québec		Ontario		Western provinces^2^	
Characteristic	*n*	%	*n*	%	*n*	%	*n*	%	*n*	%
Geographical region	226		26	11	45	20	82	36	73	32
Age										
≤25	111	49	12	46	17	38	44	54	38	52
26–30	34	15	10	38	4	9	12	15	8	11
31–40	38	17	3	11	9	20	15	18	11	15
>40	43	19	1	4	15	33	11	13	16	22
Gender										
Woman	168	74	19	73	28	62	67	82	54	74
Man & other identities	58	26	7	27	17	38	17	18	19	26
Veterinary experience										
0	148	65	24	92	20	44	53	65	51	70
1–15	35	15	1	4	10	22	18	22	6	8
16–30	20	9	0	0	7	16	5	6	8	11
>30	23	11	1	4	8	18	6	7	8	11
Graduate degree										
Yes	51	23	7	27	14	31	19	23	11	15
No	175	77	19	73	31	69	63	77	62	85
Role										
Vet dairy cows >50%	55	24	2	8	24	53	19	23	10	14
Vet dairy cows <50%	23	10	0	0.0	1	2	10	12	12	16
Vet student	148	66	24	92	20	45	53	65	51	70

^1^Atlantic provinces include Newfoundland and Labrador, Nova Scotia, New Brunswick, and Prince Edward Island.

^2^Western provinces include Manitoba, Saskatchewan, Alberta, and British Columbia.

### The role of a veterinarian

**Figure 1:**
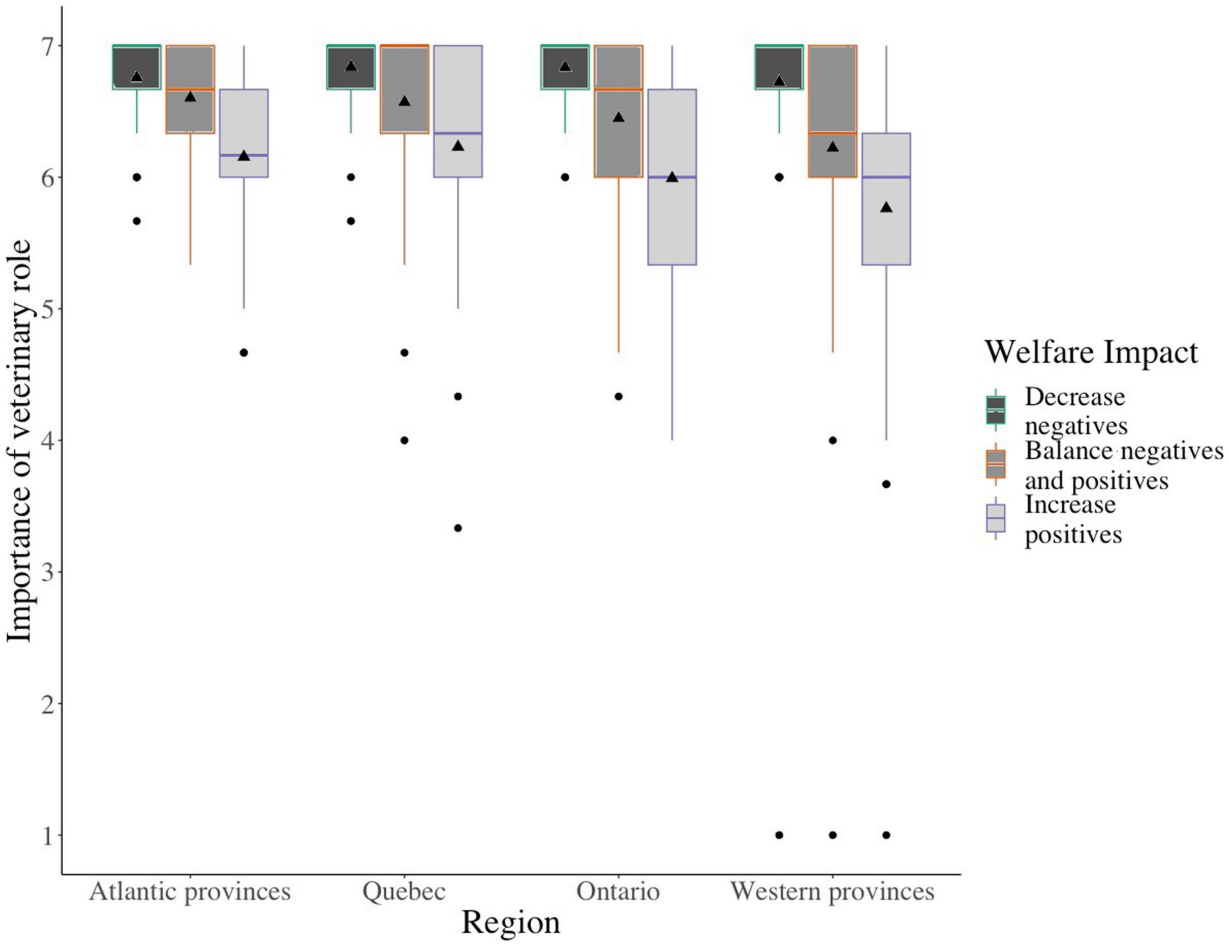
Veterinarians’ and veterinary students’ (*n* = 226) scores on a 7-point scale for three statements on the importance of the role of a veterinarian to minimize negative experiences, encourage a balance of positive and negative experiences, and encourage positive experiences for dairy cows. Score 1 is strongly disagree important and 7 is strongly agree important. The line indicates the median, triangle indicates mean, box indicates interquartile range, minimum whisker indicates Q1-1.5*IQR, maximum whisker indicates Q3 + 1.5*IQR, circles indicate outliers. Results are shown separately for participants from different geographical regions of Canada.

The final model retained region, welfare impact, and the interaction of region with welfare impact as fixed effects. The outcome variable is composed of 3 separate but related groups of questions. The model included a repeated statement to account for within-participant variation. There were small differences in participants’ scores for the importance of the role of a veterinarian in the experiences of dairy cows (*F_2,444_* = 143.64, *p* < 0. 01). Practices to minimize negative experiences were most important (mean ± SE; 6.8 ± 0.03), encouragement for a balance of positive and negative experiences was less important (6.4 ± 0.05), and encouragement of positive experiences scored lowest (6.0 ± 0.06). Geographical region explained some of the variation (*F_3,222_* = 2.70, *p* = 0.05); participants from Western provinces gave lower scores (6.2 ± 0.07) than participants from Québec (6.5 ± 0.06). Participants from the Atlantic provinces and Québec rated the balance of positive and negative experiences and encouraging positive experiences alone higher than those who lived in Western provinces (*F_6,444_* = 2.46, *p* = 0.02; Figure 1). We found no associations of role, gender, graduate training, or years of veterinary practice experience with the scores for the importance of the role of a veterinarian in each of the three areas.

**Figure 2:**
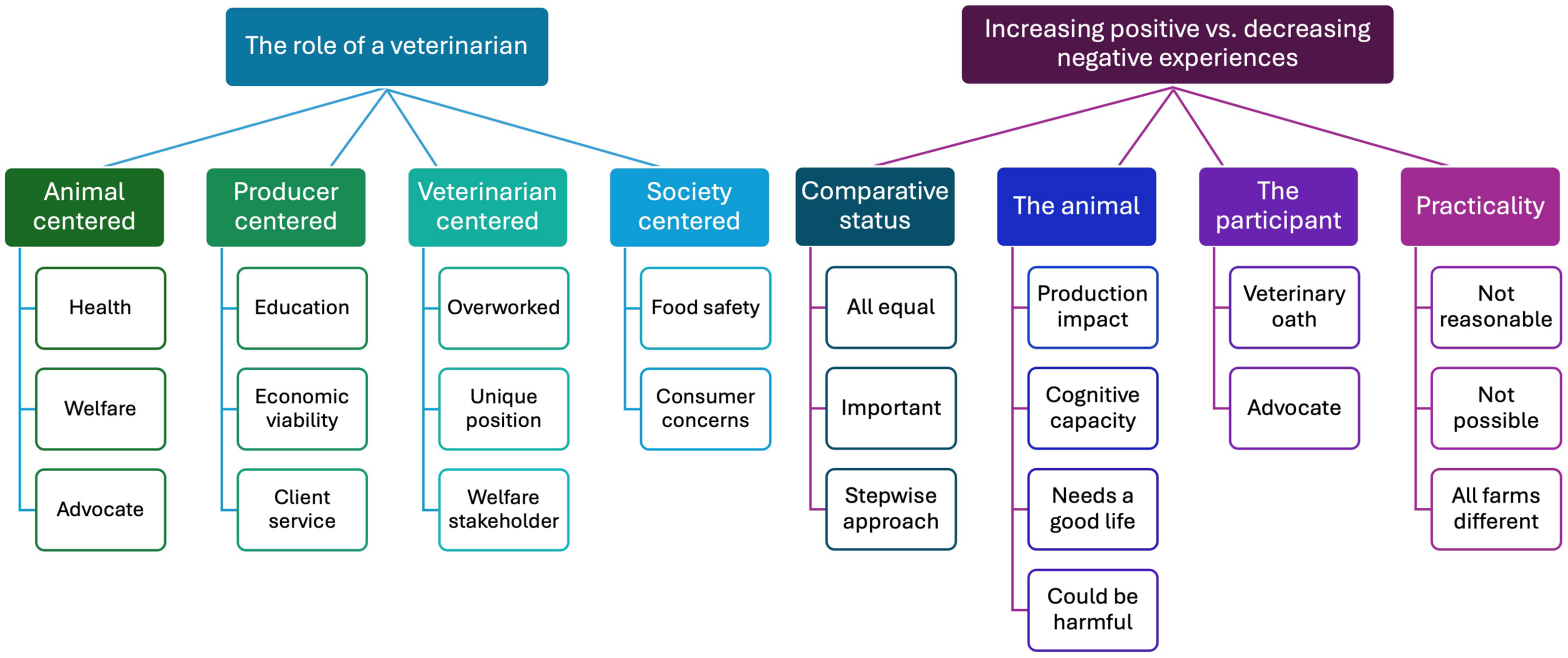
Thematic map outlining the themes and subthemes of data analysis by qualitative description generated from Canadian veterinarians and veterinary students (*n* = 226).

On average 38 ± 2 words were provided by participants to explain the role of a veterinarian regarding the experiences of dairy cows. Four themes were identified from these explanations, centered on: (1) the animal (used by *n* = 166 [61%] participants), (2) the producer (*n* = 80 [29%]), (3) the veterinarian (*n* = 17 [6%]), and (4) society (*n* = 12 [4%]) (Figure 2). Some responses referenced multiple themes (61.1%), so the total number of themes referenced was greater than the number of participants. There were 6.5% of explanations that could not be classified (for example: “no explanation required” or “N/A”).

In comments related to the animal centered theme, some participants referenced the role of veterinarian to improve cow health by minimizing disease (e.g., “*To ensure good health and treatment of disease*” WV376), pain (e.g., “*We have a higher responsibility to address pain…*” WS51) or distress (e.g., “*…minimize negative affective states more than maximize positive ones*” OS59). Others described the role to improve cow welfare. Participant WS68 stated: “*Our role is to promote welfare in our animal patients, and I think morally it is the right thing to do to ensure that these animals…live as good a life as possible*.” A third conception of this theme was that veterinarians were advocates for animals (e.g., *“[Veterinarians] have to be the voice of the animals. Use our knowledge and work experience to improve their care and well-being.*” OV287). Participants were not monolithic in their construction of the veterinary role as 35% provided multiple ideas related to the animal centered theme.

“*The veterinarian is the dairy cow’s advocate. We have the responsibility to ensure stress and pain are minimized, while also striving to provide a good quality of life for dairy cattle. Our role extends beyond simply treating and preventing disease.*” (WS99).

The producer-centered theme described the role of a veterinarian to educate producers on best management practices (e.g., “*Veterinarians are a trusted source of information for dairy producers [and] an important part of the farm team*” WV158). Some participants described how producer education surrounding “*antimicrobial stewardship and zoonotic disease transmission…*” OV3, or “*…to advocate at a policy level*” WS67, could influence welfare beyond their client and permeate the dairy industry. Other participants described the role of a veterinarian as striking a balance between animal and producer needs. Participant OS22 stated: “*I believe our role as dairy veterinarians involves promoting the physical and social well-being but also satisfying the needs of our employer, the dairy producer.*” The improved economic “*productivity*” OS22, or “*success*” OV2, of client farms was also seen as a role of the veterinarian. Participant OS138 explained:

“*As veterinarians we also have duties to our clients who are trying to make a living. Sometimes practices that would be best for cattle welfare [are] in direct conflict with our clients’ ability to have profitable businesses. For example, taking calves away from their mothers at birth.*”

Participants who discussed the veterinary profession described its role as complex (e.g., “*The role is multi-faceted and challenging. It encompasses animal health, medicine and welfare along with human interactions that require extensive finesse*” OV9), specialized (e.g., “*Vets are in a unique position to help effect change and have the knowledge to know we need to do better*” WS32) and unique (e.g., *“[Cows] should have as positive life as possible and veterinarians are in a unique position to promote that approach to dairy management*” WS40). However, some participants expressed concerns regarding “*…limits of [veterinary] training and knowledge in certain areas [of welfare]*” OV169, and “*it does not seem practical [for veterinarians to promote positive experiences] given the shortage of vets and the current pressures and mental health issues facing these professionals*” OV3. Others viewed the veterinary role as a stakeholder or team member who can facilitate compromise. Participant WS330 elaborated:

“*Veterinarians are one of the only influences on a dairy cow’s life that is not directly linked to economics and must advocate for the well-being and health even if it comes at an economic cost. Other parties involved and the veterinarian must work together to find solutions that are both economical and in the best interest of the cows. Both factors are important, but the veterinarian needs to come in at an angle that will benefit both*.”

The society-centered theme described how the role of a veterinarian served to further broader human interests, but these interests were always combined with other themes. Animal and human health appear together: “*We swear an oath to promote animal health and welfare, relieve suffering and protect the public and environment; that’s our job*” WV294, or “*Veterinarians should uphold animal welfare, animal health and public health*” WS185. Some participants, like WV273, were more specific and discussed food safety in conjunction with animal health and welfare, “*Promote animal health and welfare, prevent suffering, [and] ensure safe animal products*.” Participants described a role to address consumer concerns through two means. The first was to educate and reassure the public (e.g., “*It is the [veterinarian’s] job to educate and inform the public on what is acceptable animal behavior and welfare…*” WS266). Other participants described client education to address consumer concerns. Participant WS208 stated:

“*I think it is a veterinarian’s duty to educate their clients on best practices for managing cow health and welfare. We are a respected profession. Our education around health and welfare makes us a trusted resource for farmers…We should be encouraging farmers to use practices that provide cows a good quality of life given the importance to the animal. There is [also] growing consumer interest in [the] quality of life [of cows, making consumer interests] important.*”

### Positive vs. negative experiences

**Figure 3:**
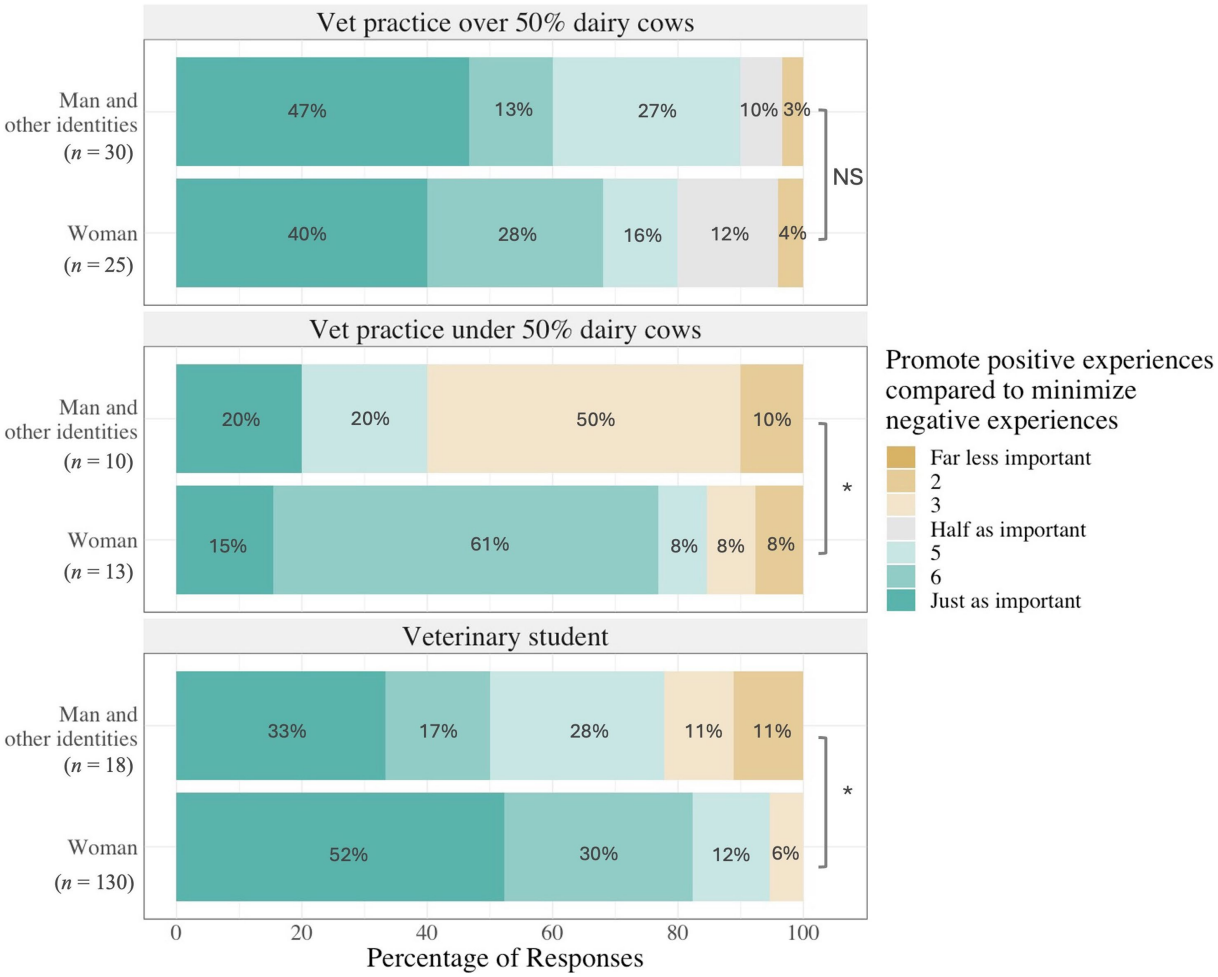
Distribution of veterinarians’ and veterinary students’ (*n* = 226) scores on a 7-point scale on the importance for veterinarians to promote positive experiences as compared to the importance to minimize negative experiences for dairy cows. Participants were asked to indicate agreement with the statement, “Overall, promoting positive experiences (e.g., natural behaviors, positive emotions, a good life) is as important as minimizing negative experiences (e.g., disease, pain, distress) for dairy cows.” Results are shown by role and separately for participants who identified as women versus men and other identities. Asterisks indicate statistically significant differences between gender within a role.

The final model retained role, gender, and role by gender interaction as fixed effects. Generally, participants indicated that promoting positive experiences was less important than mitigating negative states (5.9 ± 0.09), and ranged between not as important (2) and just as important (7). Some of the variation was explained by two demographic variables (Table 2). There was an interaction between gender and role, with participants identifying as women placing greater importance on positive vs. negative experiences for dairy cows unless they were veterinarians for whom dairy cows made up more than half of their practice (*F_2,220_* = 3.69, *p* = 0.03; Figure 3). We found no association of geographical region, graduate training, or years of veterinary practice experience with the relative importance of promoting positive versus reducing negative experiences for cows.

**Table 2:** Variables associated with the score assigned by veterinarians and veterinary students (*n* = 226) for the relative importance of promotion of positive experiences for dairy cows versus reduction of negative experiences.

Characteristic	Response	Relative importance of positive vs. negative experiences (mean ± SE)
Gender	Woman (*n* = 168) Man & other identities (*n* = 58)	6.1 ± 0.09 5.4 ± 0.21
Role	Vet dairy cows >50% (*n* = 55) Vet dairy cows <50% (*n* = 23) Vet student (*n* = 148)	5.8 ± 0.17 4.9 ± 0.36 6.1 ± 0.10

Scores are on a 1 to 7 Likert scale with 1 meaning promotion of positive is far less important and 7 meaning just as important as reduction of negative experiences for cows.

Participants provided 27 ± 1 words to explain the relative importance of increasing positive vs. decreasing negative experiences. Four themes were identified from the data, centered on: (1) frameworks to compare positive and negative status (used by *n* = 85 [39%] participants), (2) impacts on the animal (*n* = 76 [35%]), (3) the participants’ views of their role (*n* = 32 [15%]), and (4) the practicality of implementation (*n* = 24 [11%]) (Figure 2). Multiple themes were present in the answers of 9% participants and 11% of responses lacked enough detail for their explanations to be classified (for example: “nothing to add” or “agree”); therefore the total number of themes referenced was less than the number of participants.

In the second theme, many participants described different frameworks used to prioritize the promotion of positive and negative experiences of the animals. Some participants discussed that positive experiences for dairy cows are useful and important (e.g., “*Positive experiences are what make life good, avoiding the negative can only yield neutrality*,” WS40). Others justified their response by questioning the essentiality of positive experiences (e.g., “*I believe positive experiences can be beneficial but not necessary…*” OS22). Some participants, like WS309, proposed equal weight be assigned to promoting positive and minimizing negative experiences, “*Not having positive enrichment or ability to perform natural behaviors does not clearly cause physical pain as lameness does, it should be looked at with equal importance as the goal is to give dairy cows the best quality of life.*” Many participants described a stepwise approach that involved the prioritization of dairy cow experiences. Participant OS334 explained that “*Minimizing negative experiences should be priority number one whereas enrichment and opportunity for positive experiences should be goal number two*.” Other participants provided additional details on the internal struggle to assess the comparative value of positive experiences for dairy cows. Participant WS105 described their rationale:

“*It’s a tough question and is debatable. Promoting positive experience is important but I would not say AS* (their emphasis) *important as minimizing negative experiences. I would rather see a herd with zero lameness, post-partum ketosis, mastitis*, etc.*, than a herd that has some of those but also allows cows to graze on pasture. It’s all very situational, and a tough question for me to answer.*”

The animal centered theme focused on the dairy cow. Some participants described the mental capacities of cows (e.g., “*…Cows are very smart and deserve enrichment in their lives.*” WS44) while others questioned these capabilities (e.g., “*…I am not aware that cows have emotions, other than wanting to feel safe and fed so I do not know what other ‘positive emotions’* (their emphasis) *we are trying to achieve*” WS63). Many participants, including WS312, described how production was positively linked to positive experiences, “*It is very important to promote positive experiences for dairy cows [because it’s] the ethical or moral thing to do and is economically beneficial.*” However, others questioned if some positive experiences could decrease production or harm the animals themselves (e.g., “*Allowing for behaviors like free access to pasture have many negative health, welfare and economic implications [including] mastitis, low milk production, heat stress, reproduction problems*” WV6). Participants also described that dairy cows should have “a good life” WS104, “a life worth living” WS71, or “a fulfilling life” WS42. Participant WS32 expressed concern about different standards of care between agricultural and companion animals:

“*Cows currently do not experience a lot of positive welfare. All animals deserve to live pain free but [also] positively engaged lives. There would be outrage if dogs were kept in tie stalls. Cows should be given the same quality of positive experiences we strive to provide our pets and ourselves*.”

Some responses that fell into the participant centered theme mentioned the veterinary oath (e.g., “*Following our veterinarian’s oath to promote [experiences] that positively impact dairy cattle health and welfare*” OV9), and ethical responsibilities or moral obligations to animals (e.g., “*It’s very important to promote positive experiences for dairy cows. Not only is it the ethical [and] moral thing to do, but it’s economically beneficial*” WS312). Many respondents described themselves as advocates for animals, “*If veterinarians have the knowledge and ability to integrate opportunities for positive experiences for cows, we should be advocating for it*” WS86. However, there was disagreement whether animal advocacy included promoting positive experiences, “*Positive animal experiences is an ethical responsibility of animal owners…*” WS117. Participant WV283 also acknowledged the competing interests faced in the agricultural production setting, “*[We] advocate the best interests of the animals and help the farmer achieve those interests to the best of their abilities*.” This theme also elucidated challenges that participants faced:

“*Although I strongly agree [with promoting positive experiences], in practice it is difficult to prioritize these needs. As a veterinarian, it is my job to advocate for these animals not only to relieve suffering but to encourage positive welfare in whatever capacity is possible while working with the farmer*” WS33.

The theme centered on practicality addressed the challenge that promotion of positive experiences may not be reasonable or possible. Participant WS69 described that “*Ideally making sure dairy cows have positive experience is just as important. However, it is more urgent to ensure that minimum welfare standards of all are met*.” Other participants cited external conditions that challenge positive welfare opportunities (e.g., “*Canada’s winter will limit natural behaviors*” WV5, “*It’s not my cow*…” OV165, “*producer space, staff, financial constraints*” WS79, and “*defining positive welfare*” WV172). Participant OV143 reflected on systemic barriers that affect the practicality of solutions, “*The code of ethics says to do no harm but unfortunately, we often are faced with [dairy housing] systems that do not allow us to practice it. Very uncomfortable industry wide changes are required to move towards more positive experiences for livestock*.” Many participants described that a single approach for the promotion of positive experiences would not be feasible for the diverse housing systems and management strategies that currently exist (e.g., “*Positive experiences can look differently in different barns, facilities and systems*” WV1). Participant WS37 integrated numerous aspects of this theme to describe their rationale:

“*Ideally we would work to provide more positive experiences to dairy cows. That being said, in the [dairy] industry this focus is not as important as preventing suffering or distress. There are only so many things you can reasonably work with [clients] and advise them to start, stop, or change. I feel that the onus to ensure negative experiences are avoided weighs more heavily than the opposite. There are issues in the industry that result in the negative experiences for cattle, and these must be addressed before turning to positive experiences, unfortunately.*”

## Discussion

When asked about the role of a veterinarian to ensure dairy cattle welfare we described and confirmed, via analysis, the three distinct *a priori* conceptions of welfare impacts used by Canadian dairy veterinarians and veterinary students: minimizing negatives, increasing positives, and creating a positive–negative balance. Overall, our participants valued these distinct welfare impacts differently. Participants identified the primary role of a veterinarian to be minimizing experiences that resulted in negative welfare for dairy cattle. This finding is supported by previous research that identified veterinarians as key sources of information on prevention and treatment of disease (20, 21), biosecurity (46), and antimicrobial use (47) which minimize negative outcomes for animals. While scored slightly lower, the veterinarian’s role was also perceived to facilitate an increase in positive experiences and to create a balance between positive and negative experiences for dairy cows. Similar theoretical pluralistic conceptions of positive welfare have been proposed in the literature (8) and are supported by our empirical findings. Novel to this study was the identification of a region by type of welfare impact association where the role of a veterinarian to promote positive experiences and promote a balanced experience was seen as less important by participants in Canada’s western provinces. It is unclear if the regional differences detected might be associated with cultural differences, predominant dairy management system, variation in veterinary curricula, or are possibly spurious. Additional studies are encouraged to determine the basis of these regional differences.

No association was found between practicing veterinarians and veterinary students regarding the perceived role of the veterinarian in ensuring dairy cattle welfare which was unexpected. Research that assessed American Veterinary Medical Association (AVMA) Council of Education accredited institutions found six (12%) offered formal courses in animal welfare in 2016 (48). The AVMA Animal Welfare Curriculum Planning Group then provided a framework to integrate animal welfare into veterinary curricula and to assess veterinary student competence (49). Given that veterinary students and recent graduates participated in this study we anticipated that their educational exposure to animal welfare training would have informed their responses. However, we did not assess variation in welfare curriculum between institutions or if the current framework aimed to educate veterinary students is sufficient to influence perceptions of animal welfare. Several veterinary students provided concerning statements that questioned the capacity of cattle to experience affective states or benefit beyond minimizing negative experiences. Given that veterinary students often inconsistently apply animal welfare concepts (30–33) future research to investigate the ability of current veterinary training methods to build competency in companion, agricultural, and wild animal welfare is encouraged.

Respondents rated minimizing negative experiences nearly 20% more important than promoting positive experiences. This finding is unsurprising given the pragmatic triage-based approach used in veterinary medicine (50, 51) and focus on physical health of dairy cattle (26–28). Many participants highlighted ethical and professional responsibilities, which were noted to focus ideally on all aspects of an animal’s welfare. However, such efforts were described as impractical due to excessive case load and demands of work primarily devoted to disease prevention and treatment. This finding is supported by research that found dairy veterinarians focused on physical health aspects of welfare at the expense of calf social requirements (29). The inability to navigate ethical and practical obligations may contribute to poor mental health among Canadian veterinarians (52). Previous research has found higher dairy farmer welfare on farms employing practices that improve cow welfare; increased autonomy with robotic milking systems (53), cow-calf-contact systems (54), and pasture-based systems (55). Additional research should explore the intersection of animal and human welfare; specifically, if and how providing improved animal welfare can also improve human welfare.

Both veterinary students and veterinarians with a low emphasis on dairy cattle who did not identify as women placed less importance on promoting positive experiences than veterinarians with a high emphasis on dairy cattle who did not identify as women. Other studies (56–58) found that attitudes toward animals were influenced by gender, which aligns with the gender differences we observed within veterinary students and veterinarians from practices with less than 50% dairy cattle. This underscores that veterinarians are not monolithic and cannot be described by a generalized construction of ‘a veterinarian’. The veterinary profession is described as having diverse societal, cultural, and individual views on animals (59) yet lacking the diversity representative of the populations they serve (60). The use of humanities and social science approaches in additional research could begin to address complex problems facing veterinary medicine such as decreasing numbers of large animal care practitioners, societal expectations for the profession, and veterinarians’ mental health (61).

The qualitative responses revealed a diversity of nuanced views. Over 60% of justifications regarding the role of a veterinarian and 35% of responses about the importance for veterinarians to promote positive versus minimize negative experiences for dairy cows mentioned the experience of the animals. Similarly, other mixed-methods research found the animals’ experiences influenced perceptions of contentious procedures (62) and ethical and regulatory oversight (56) of animal experimentation. We were surprised that even more responses did not include the experience of the animal given our survey topic and could demonstrate challenges faced by veterinary professionals. Research suggests veterinarians face burnout (52), poor mental health (52, 63, 64) which impact their perceived ability to provide medical care (65) and meet the needs of their clients (66).

Multiple themes were described in less than 10% of the relative importance responses. Taken with the quantitative results, the use of singular themes demonstrates specific participant rationale to justify why the reduction of negative experiences takes priority over promoting positive experiences. Conversely, over 60% of responses regarding a veterinarian’s role to improve welfare had multiple themes, which demonstrates a complex conception of this role, integrating aspects of the animal, producer, veterinary profession, and society. Likewise, other studies have found that veterinarians have complex conceptions of diagnosis (22), practice recruitment and retention (67), and institutional transparency (68). Our results highlight the strength of mixed-methods studies to detect nuanced participant reasoning and inform additional research.

There are limitations with the methodology of our survey. We recruited Canadian veterinarians and veterinary students and our ability to generalize beyond this group is limited. Nevertheless, other research on animal welfare issues across multiple developed countries found the direction of responses was consistent among countries while the intensity of response varied (69). We acknowledge the potential for sampling basis as a result of our email recruitment strategy. Respondents may have been more likely to participate if they had an interest in dairy cattle welfare. While our qualitative answers provided a diversity of views from respondents, additional studies should employ methods to engage those who did not participate (70), and could include a participatory study design to drive involvement inside the veterinary and veterinary student populations. We arranged some of our questions into *a priori* conceptions of welfare and did not provide participants the opportunity to define animal welfare. Given that other research has shown veterinarians differ in how they prioritize dairy welfare concerns (29, 71), initial participant definitions could have affected the comparison of the promotion of positive experiences versus decreasing negative experiences. We specifically suggest future research that uses qualitative methods to identify subtle differences in the conception of positive welfare among veterinarians. The participants in our study were mostly younger, women, veterinary students from Ontario and the western provinces. The convenience sample provided access to the study population via email and resulted in similar response rates from veterinarians vs. veterinary students and among regions but may not have been distributed to all potential participants. Although we did not have data on the gender breakdown of the target population, other research (72–74) supports that the gender distribution of our respondents aligns with the target population.

This study described the perceived role of the veterinarian in the promotion of dairy cattle welfare. These results describe different conceptions and valuations of how the veterinarian should influence animal welfare. There were small differences in emphasis on efforts to minimize negative, promote positive, and balance positive and negative experiences for cows. We conclude that veterinarians are favorably disposed to positive aspects of welfare for dairy cows but are more focussed on avoidance of negative aspects of welfare.

## Data availability statement

The datasets presented in this study can be found in online repositories. The names of the repository/repositories and accession number(s) can be found below: All data, code, and materials used in the analysis are available in Borealis, the Canadian Dataverse Repository at: https://doi.org/10.5683/SP3/EQN2G3.

## Ethics statement

The studies involving humans were approved by University of Guelph, Research Ethics Board. The studies were conducted in accordance with the local legislation and institutional requirements. The participants provided their written informed consent to participate in this study.

## Author contributions

MB: Conceptualization, Funding acquisition, Investigation, Methodology, Project administration, Visualization, Writing – original draft, Writing – review & editing. DH: Writing – review & editing. SL: Funding acquisition, Writing – review & editing. DK: Conceptualization, Funding acquisition, Supervision, Writing – review & editing.

## Footnotes

^1^ https://doi.org/10.5683/SP3/EQN2G3
